# Identification and Validation of Small-Gatekeeper Kinases as Drug Targets in *Giardia lamblia*

**DOI:** 10.1371/journal.pntd.0005107

**Published:** 2016-11-02

**Authors:** Kelly M. Hennessey, Tess R. Smith, Jennifer W. Xu, Germain C. M. Alas, Kayode K. Ojo, Ethan A. Merritt, Alexander R. Paredez

**Affiliations:** 1 Department of Biology, University of Washington, Seattle, Washington, United States of America; 2 Division of Allergy and Infectious Diseases, Center for Emerging and Re-emerging Infectious Disease (CERID), University of Washington, Seattle, Washington, United States of America; 3 Department of Biochemistry, University of Washington, Seattle, Washington, United States of America; McGill University, CANADA

## Abstract

Giardiasis is widely acknowledged to be a neglected disease in need of new therapeutics to address toxicity and resistance issues associated with the limited available treatment options. We examined seven protein kinases in the *Giardia lamblia* genome that are predicted to share an unusual structural feature in their active site. This feature, an expanded active site pocket resulting from an atypically small gatekeeper residue, confers sensitivity to “bumped” kinase inhibitors (BKIs), a class of compounds that has previously shown good pharmacological properties and minimal toxicity. An initial phenotypic screen for biological activity using a subset of an in-house BKI library found that 5 of the 36 compounds tested reduced trophozoite growth by at least 50% at a concentration of 5 μM. The cellular localization and the relative expression levels of the seven protein kinases of interest were determined after endogenously tagging the kinases. Essentiality of these kinases for parasite growth and infectivity were evaluated genetically using morpholino knockdown of protein expression to establish those that could be attractive targets for drug design. Two of the kinases were critical for trophozoite growth and attachment. Therefore, recombinant enzymes were expressed, purified and screened against a BKI library of >400 compounds in thermal stability assays in order to identify high affinity compounds. Compounds with substantial thermal stabilization effects on recombinant protein were shown to have good inhibition of cell growth in wild-type *G*. *lamblia* and metronidazole-resistant strains of *G*. *lamblia*. Our data suggest that BKIs are a promising starting point for the development of new anti-giardiasis therapeutics that do not overlap in mechanism with current drugs.

## Author Summary

The eukaryotic protozoan *Giardia lamblia* is the most commonly reported intestinal parasite worldwide. Current treatments used to treat giardiasis include metronidazole and other nitroimidazole derivatives. However, emergence of metronidazole-resistance strains and adverse reactions to the treatments suggest that alternative therapies against giardiasis are necessary. Here we identify a set of protein kinases in the *Giardia* genome that have an atypically small amino acid residue, called the gatekeeper residue, in the ATP binding pocket. Small gatekeeper residues are rare in mammalian kinases. We investigated whether this subset of kinases is necessary for parasite growth and proliferation and, if so, could they be targeted with a class of compounds called bumped kinase inhibitors (BKIs), designed to exploit the enlarged active site pocket made accessible by the small gatekeeper amino acid. Morpholino knockdown of two of the small gatekeeper kinases produced a distinctive phenotype characterized by defective cytokinesis. This phenotype was mimicked in *Giardia* cells treated with our most potent BKI. These results suggest that BKIs may be developed to selectively target small gatekeeper kinases in *Giardia lamblia* to provide a novel treatment option for giardiasis.

## Introduction

*Giardia lamblia* is the most commonly reported intestinal protozoan parasite and the cause of giardiasis, a gastrointestinal illness resulting in diarrhea, nutrient malabsorption, vomiting, and weight loss [1]. It infects approximately 280 million people worldwide [2,3,4]. This disease contributes to the global health burden of diarrheal diseases that collectively constitute the second-leading cause of death in children under five years old [3,4]. Infection can also cause developmental delays and failure to thrive [5]; as few as 3 occurrences (>2 weeks duration) of diarrheal disease per year during the first 2 years of life is associated with reduced height (approximately 10 cm) and intelligence quotient score (10 points) by 7–9 years of age [6]. *G*. *lamblia* has a simple life cycle consisting of two forms, the binucleate flagellated trophozoites and the tetranucleate infective cysts. Cysts are the environmentally resistant forms responsible for transmission of the disease [1].

First choice therapeutic options are limited to metronidazole and chemically related nitroimidazole drugs. These compounds are prodrugs whose reduction to reactive radicals is mediated intracellularly by pyruvate: ferredoxin oxidoreductase and other enzymes involved in anaerobic metabolism. Resistance can occur in up to 20% of clinical presentations, primarily due to down-regulation or mutation of these activating enzymes [7,8]. The toxic intermediates cause DNA damage in *Giardia* trophozoites [9], and attack protein sulfhydryl groups non-specifically. Even when infection is cleared, pathophysiological changes in the gut may persist, severely impacting quality of life [3,8]. Consequently, there is an increasing need to develop alternative drugs to treat giardiasis.

To address this need, we have combined a structure-based approach with targeted phenotypic screening to jointly identify and validate a class of potential protein targets in *Giardia* and a corresponding class of drug-like molecules that attack them. This approach takes advantage of an in-house library of protein kinase inhibitors based on a limited number of chemical scaffolds, developed in the course of previous work to optimize potency, pharmacological properties, and selectivity for inhibition of CDPK (Calcium Dependent Protein Kinase) homologs in several apicomplexan pathogens [10,11]. A primary structural determinant of target selectivity in this library is the fortuitous presence of an atypically small gatekeeper residue in the active site of the target CDPKs [12,13]. The presence of a small amino acid at the gatekeeper position creates a much larger effective pocket than is found in the majority of protein kinases [14], allowing inhibition by compounds that are too large to be accommodated in a typical kinase active site. Compounds from this library have been shown to have minimal cytotoxicity against human cells, consistent with selective activity disfavoring inhibition of human kinases. Several have shown promise in animal trials for anticoccidial efficacy [15,16]. While design of the 400+ compounds in our BKI (bumped kinase inhibitor) library was biased toward optimal selectivity for CDPK homologs, all library compounds are expected to preferentially inhibit small gatekeeper kinases.

Protein kinases in general constitute an attractive class of molecular targets for drug discovery, distinct from the targets of existing anti-giardiasis drugs. Of the 278 protein kinases identified in the *G*. *lamblia* genome (strain WB), 80 kinases form a core kinome while the remaining 198 constitute a massively expanded family of NEK kinases [17]. The core kinome contains 80 kinases from 49 families that are recognizably also present in higher eukaryotes. It contains 19 families that have no recognized homologs outside of *Giardia* [17]. Where direct homologs can be identified, the average sequence identity between *Giardia* and human homologs is roughly 40% [17]. Thus, individual *Giardia* kinases are in general expected to show extensive sequence and structural differences to their human homologs, if any, facilitating development of selective inhibitors. Furthermore, the greatly reduced number of core kinome classes, each containing no more than three members in the *G*. *lamblia* genome, suggests that gene loss in these parasites has pared the remaining core kinome down to a near-minimal set of essential proteins.

The huge expansion of NEK kinase sequences in the *Giardia* genome stands in contrast to the reduction in size of the core kinome. Similar, though less extreme, expansions of the NEK kinase group have been found in ciliates and excavates [18,19]. The total of 198 NEK homologs in the *G*. *lamblia* genome may be compared to a single NEK homolog in yeast and 11 in humans. Many of the *Giardia* NEK sequences are highly variable across strains, and roughly two-thirds are inferred to be catalytically inactive due to the loss of conserved catalytic residues in the canonical active site [17]. Nevertheless, among the catalytically active *Giardia* NEK kinases some are likely to carry out biologically essential phosphorylation and hence to constitute valid drug targets. In other eukaryotes, NEK kinases are involved in cell cycle control [20,21]. Two *Giardia* NEK kinases have been shown to be active in mitosis and excystation [22]. It is worth noting that two trypanosomal NEK kinases, one of them coincidentally possessing a small gatekeeper residue, have been suggested as drug targets in *Trypanosoma brucei* [23].

While the *G*. *lamblia* kinome contains no CDPK homologs, it does contain multiple genes encoding kinases with small gatekeeper amino acid residues in their ATP binding sites, creating a potential opportunity to design specific ATP-competitive inhibitors that are highly selective for one or more parasite kinases relative to all human kinases. Using reverse genetics, we show that some of these kinases are essential for *Giardia* trophozoite proliferation. In addition to providing insight into the role of these newly described kinases, we also show that they are targets of some BKIs. Our results constitute the first steps toward further development of BKIs into an effective alternative treatment for giardiasis.

## Methods

### Bioinformatics and target selection

The sequence of the TgCDPK1 kinase domain core was used as a BLASTP probe of *Giardia* strain WB sequences in GiardiaDB. This search returned 232 hits in total. Of these, 121 had E scores < 2x10^-4^ and within this set the initial alignment for eight *Giardia* kinases indicated a glycine, alanine, serine, or threonine gatekeeper residue. These eight, all with E scores < 4x10^-24^, were selected for the initial evaluation presented here, as their substantial sequence similarity to TgCDPK1 was expected to indicate higher likelihood of sensitivity to the existing BKI library compounds.

Subsequently, ClustalW/HMMER multisequence alignment was used to match all 232 sequences against TgCDPK1 residues 110–133, corresponding to the β-hairpin containing the gatekeeper residue itself and residues contributing to the active site pocket. Four additional putative small-gatekeeper kinases were identified from this search and earmarked for eventual characterization paralleling the work reported here. Finally, we noted that 15 putative kinase ORFs annotated as active in the tabulation by Manning [17] were not recovered by either of our probes. These were matched individually to the nearest homolog with a representative structure in the PDB in order to identify the gatekeeper residues with certainty.

### Parasite culture

*G*. *lamblia* wild-type strains *WBC6* (ATCC 50803), *713* and metronidazole-resistant *713-M3* cells [24] (supplied by the L. Eckmann lab, UC San Diego School of Medicine), were grown in TYI-S-33 medium supplemented with 10% bovine serum and 0.05 mg/mL bovine bile [25]. Cells were cultured at 37° under hypoxic conditions using 15 mL polystyrene screw-cap tubes (Corning Life Sciences DL).

### *In vivo* compound screen assays

Preliminary phenotypic screening for inhibitory effects of 36 compounds from our focused BKI library [26] against the trophozoite stage of *G*. *lamblia* was carried out at a final concentration of 5 μM. This library subset was chosen to include multiple chemical scaffolds and both large and small substituents at the R1 and R2 scaffold positions [10,27,28]. Compounds were screened in 48-hour growth assays in 96-well microtiter plates. Each compound (150 μL) was added to 150 μL of well-suspended diluted parasites (~20,000 cells/mL) and incubated in anaerobic BD GasPak Bio-Bags (Becton Dickinson, San Jose, California). After 48 hours, 96-well plates were placed on ice for 30 minutes, fixed with 0.64% paraformaldehyde, and thoroughly resuspended and counted with a MoxiZ Coulter counter (Orflo Technologies, Hailey, ID.)

### Vector construction, transfection, and protein expression

To generate endogenously tagged small gatekeeper kinases, the putative kinase-coding genes were amplified from genomic DNA by PCR and cloned into the pKS_3HA_Neo vector [29]. Primer sequences and restriction enzymes are shown in S1 Table. PCR amplifications were performed using iProof DNA polymerase (Bio-Rad). Typically, an amplicon of ~ I kb in length that lacked the start codon was cloned in frame to a C-terminal triple-hemagglutinin epitope tag (3xHA) into the pKS_3HA_Neo plasmid. The plasmids were linearized by the enzyme reported in S1 Table and ~5 μg of DNA was used to transform wild-type *Giardia*. Transformants were selected with G418 at 40 μg/mL.

For protein expression, the complete coding region of each protein kinase was PCR amplified from *Giardia* genomic DNA. PCR amplicons were cloned into the ligation independent cloning (LIC) site of (MBP)-AVA0421 expression vector and validated by sequencing [30]. Recombinant proteins were expressed in *E*. *coli* BL21 (DE3), Invitrogen, Carlsbad, CA) using Studier auto-induction protocols at 20°C [31]. Recombinant protein purifications were performed as previously described [26].

### Western blot analysis

*Giardia* trophozoites were harvested after chilling cultures on ice for 30 minutes. After detachment, cells were pelleted at 700xg, washed once in HBS (HEPES buffered saline), then resuspended in 300 μL of lysis buffer (50 mM Tris pH 7.5, 150 mM NaCl, 7.5% Glycerol, 0.25 mM CaCl_2_, 0.25 mM ATP, 0.5 mM DTT, 0.5 mM PMSF, 0.1% Triton X-100, Halt 100X Protease Inhibitor Cocktail (ThermoFisher Scientific), then sonicated. The lysate was cleared by centrifugation at 10,000xg for 10 minutes at 4°C and then boiled in 2x Laemmli Sample Buffer (Bio-Rad). After SDS-PAGE, samples were transferred to PVDF membrane (Immobilon-FL) following the manufacturers’ directions. Primary polyclonal rabbit anti-giActin 28PB+1 [32] and monoclonal anti-HA mouse HA7 antibodies (IgG1; Sigma-Aldrich) were diluted 1:2500 in blocking solution (5% dry milk, 0.05% Tween-20 in TBS). Secondary anti-mouse Alexa-555 and anti-rabbit Alexa-647 antibodies were used. Horseradish peroxidase-linked anti-mouse or anti-rabbit antibodies (Bio-Rad) were used at 1:7,000. Multiplexed immunoblots were imaged on a Chemidoc MP (Bio-Rad) and signals were quantitated using ImageJ [33].

### Immunofluorescence analysis

*G*. *lamblia* cells were pelleted at 500xg at room temperature, the pellet and remaining attached cells were fixed in PME (100 mM Pipes pH 7.0, 5 mM EGTA, 10 mM MgSO_4_) plus 0.025% Triton X-100, 100 μM MBS, and 100 μM EGS for 30 minutes at 37°C. Cells were again pelleted, washed, resuspended with PME, and adhered to poly-L-lysine (Sigma-Aldrich) coated coverslips. Cells were permeabilized in PME + 0.1% Triton X-100 for 10 minutes then washed 2X with PME + 0.1% Triton X-100 and blocked for 30 minutes in PMEBALG (PME + 1% BSA, 0.1% NaN_3_, 100 mM lysine, 0.5% cold water fish skin gelatin (Sigma Aldrich, St. Louis, MO) [32]. Cells were stained with rabbit anti-giActin antibody 28PB+1 [32] and mouse monoclonal anti-HA (Clone HA7, Sigma-Aldrich) both diluted 1:125 in PMEBALG and incubated overnight. After three subsequent washes with PME + 0.05% Triton X-100, cells were incubated in secondary antibodies Alexa-488 goat anti-mouse and Alexa-555 goat anti-α-rabbit (Sigma-Aldrich, St. Louis, MO) (diluted 1:125 in PMEBALG) for 1 hour [32]. Cells were washed three times with PME + 0.05% Triton X-100. The coverslips were mounted with ProLong Gold anti-fade plus DAPI, (Thermo Fisher Scientific, Rockford, IL). Fluorescence deconvolution microscopy images were collected as described [34]. A minimum of 150 cells were examined and 30 imaged per experiment.

### Morpholino knockdown and growth assays

Trophozoites were cultured to confluency, iced for 30 minutes to detach, spun down (500xg for 5 minutes) and media was replaced with 1.0 mL fresh *Giardia* growth medium. Cells and cuvettes were chilled on ice. Lyophilized morpholinos listed in S1 Table (Gene Tools, LLC, Philomath, OR) were resuspended in sterile water and 30 μL of a 100 mM morpholino stock was added to 300 μL of cells in a 4 mm cuvette. We used Gene Tools, LLC standard morpholino as a negative control. Cells were electroporated (375V, 1000 μF, 750 Ohms, GenePulser Xcell, Bio-Rad, Hercules, CA). Cells were transferred to fresh media and incubated 4 hours at 37°C to allow cells to recover. Cells were then iced for 30 minutes, counted and diluted to 20,000 cells/mL. Aliquots were counted every 12 hours over 48 hours. All cell counting was done using a Coulter counter (MoxiZ). Three independent replicates of each cell line and control were analyzed for each time point. Quantification of protein expression was determined at the 24-hour time point by the Western blot assay described above.

### Attachment assays

Trophozoites were cultured and treated with morpholinos as described above. After 4 hours of recovery, cells were iced for 30 minutes, counted, and then diluted to 20,000 cells/mL. After 48 hours, the media was decanted into a fresh tube and replaced with 1XPBS (phosphate buffered saline). Both tubes were placed on ice to detach or prevent attachment. The cells from each group were then counted, using a Coulter counter (MoxiZ), as above.

### Thermal shift assays

Thermal shift assays on purified *E*. *coli* expressed Gl50803*_*8445 and Gl50803*_*16034 proteins were performed as described previously [35].

### EC50 compound screen assays

EC50 assays were performed on wild type *Giardia* trophozoites to determine the potency of each BKI that elicited a temperature shift in the thermal shift assays. *Giardia* trophozoites were harvested after chilling cultures on ice for 30 minutes. Three-fold serial dilutions of compounds from 10 μM to 1.524 nM concentrations were created in fresh *Giardia* growth media and growth assays were set up in 96-well microtiter plates as above. Growth was assayed after 48-hours and EC50 values were determined using gnuplot.

### Accession numbers

**Genes/proteins**
**8445:** UniProtKB—A8BXP9 (A8BXP9_GIAIC)
**9421:** UniProtKB—A8BDH0 (A8BDH0_GIAIC)
**9665:** UniProtKB—A8BZJ9 (A8BZJ9_GIAIC)
**11364:** UniProtKB—E2RTY0 (E2RTY0_GIAIC)
**12148:** UniProtKB—A8BW54 (A8BW54_GIAIC)
**13215:** UniProtKB—A8B2W4 (A8B2W4_GIAIC)
**16034:** UniProtKB—A8BHW1 (A8BHW1_GIAIC)
**17368:** UniProtKB—A8BQY4 (A8BQY4_GIAIC)

## Results

### Basis for BKI selective activity

BKIs originated conceptually with the observation that most protein kinases will not tolerate ATP analogs containing a “bump” on the ATP purine ring. This restriction arises because the sidechain of a specific residue, the gatekeeper, limits the volume available in the active site to accommodate such chemical modification of the substrate. This observation was exploited, notably by the Shokat group [26,36], to probe the function of individual kinases *in vivo* by introducing an engineered variant in which the naturally occurring gatekeeper was replaced by a smaller residue, making the engineered kinase uniquely competent to recognize bumped ATP analogs. The precision of this technique is possible because naturally occurring kinases with gatekeepers smaller than threonine (i.e. glycine, serine, alanine) are extremely rare [14]. Threonine is less rare as a gatekeeper residue although still much less common in the human kinome than larger gatekeepers, particularly methionine.

**Fig 1:**
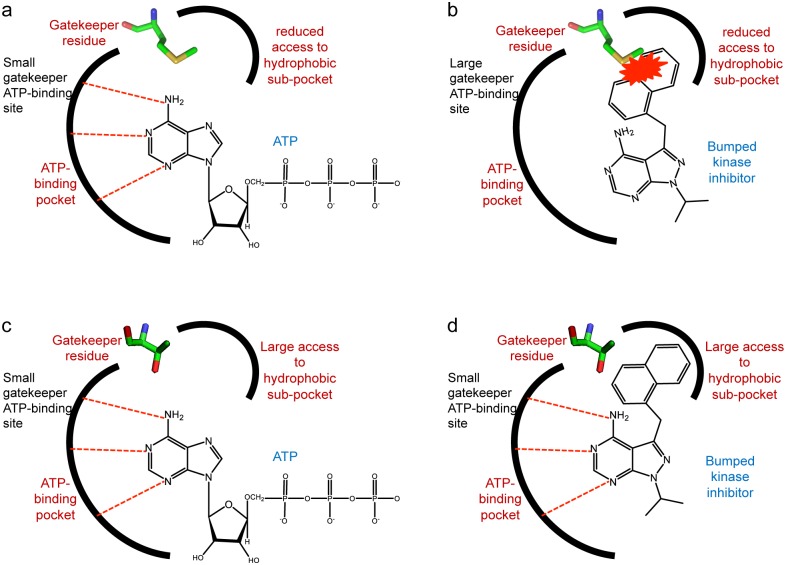
Structural differences between small gatekeeper ATP binding sites and large gatekeeper ATP binding sites. Structural differences form the basis for BKIs to selectively act on the *Giardia* kinases described in this study. **(a)** The ATP binding pocket of protein kinases typically contains a large amino acid gatekeeper residue (e.g. methionine). **(b)** BKIs do not bind to the ATP binding site of typical kinases because of a clash between the “bump” and the side chain of the gatekeeper. **(c)** A small amino acid in the gatekeeper position has no implications for ATP access to the ATP binding site. **(d)** In contrast, the small gatekeeper residue permits the BKI “bump” to extend into the hydrophobic subpocket and outcompete ATP.

Notwithstanding their rarity in the human kinome, small gatekeeper kinases are found naturally in the genomes of various protozoa. Individual small gatekeeper kinases have been characterized as potential targets for drug development against eukaryotic pathogens such as *T*. *brucei* [23], *T*. *gondii* [37], *C*. *parvum*, and *P*. *falciparum* [12,26,27]. In the case of apicomplexan targets, BKIs have been designed for high selectivity relative to all human kinases, including human threonine gatekeeper kinases, by simultaneously exploiting the gatekeeper-mediated restriction (Fig 1) and the geometry of the ribose binding pocket in the specific target kinase [38].

### *Giardia lamblia* has multiple small gatekeeper kinases

A genome wide search of the *Giardia lamblia* genome for genes encoding kinases with small amino acid gatekeeper residues was performed using the core kinase domain of *Tg*CDPK1 as the sequence template. *Tg*CDPK1 was chosen because it was a primary target guiding the assembly of the BKI library used in this study [13]. BLASTP results identified eight putative kinase-coding sequences with small gatekeeper residues. The gatekeeper residues included a threonine in kinases *Gl50803_8445*, *Gl50803_9665*, *Gl50803_11364*, *Gl50803_16034* and *Gl50803_17368*, a glycine in *Gl50803_13215*, an alanine in *Gl50803_12148* and a serine in *Gl50803_9421*. The Ala-gatekeeper sequence (*Gl50803_12148*) belongs to a catalytically inactive NEK kinase [17] and was not pursued further.

**Fig 2:**
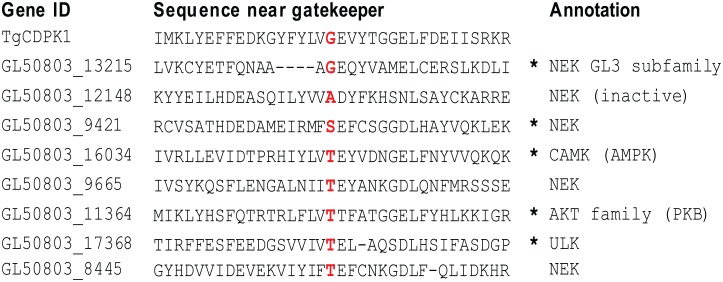
Initial set of eight putative *Giardia lamblia* small gatekeeper kinases. BLASTP against the *Giardia* genome database using the *T*. *gondii* CDPK1 core kinase domain as a probe found 8 kinases with substantial sequence similarity (E < 10^−24^) to the probe whose alignment indicated a small gatekeeper residue (threonine or smaller). Sequences near the small gatekeeper are shown with small gatekeeper amino acids in red.

Of the seven small gatekeeper kinases predicted to be sensitive to BKI inhibition, four (*Gl50803_8445*, *Gl50803_9421*, *Gl50803_9665*, and *Gl50803_13215*) are *Giardia*-specific NEK kinases. Based on sequence homology, kinase *Gl50803_17368* is inferred to be a member of the ULK family. *Gl50803_16034* is inferred to be a member of the CAMKL/AMPK family (Fig 2). *Gl50803_11364* is one of the few *G*. *lamblia* kinases that have been previously investigated. This AKT family kinase is differentially expressed in encystation compared to the trophozoite stage but its regulatory function is unknown [39].

### BKIs inhibit *Giardia* growth

**Fig 3:**
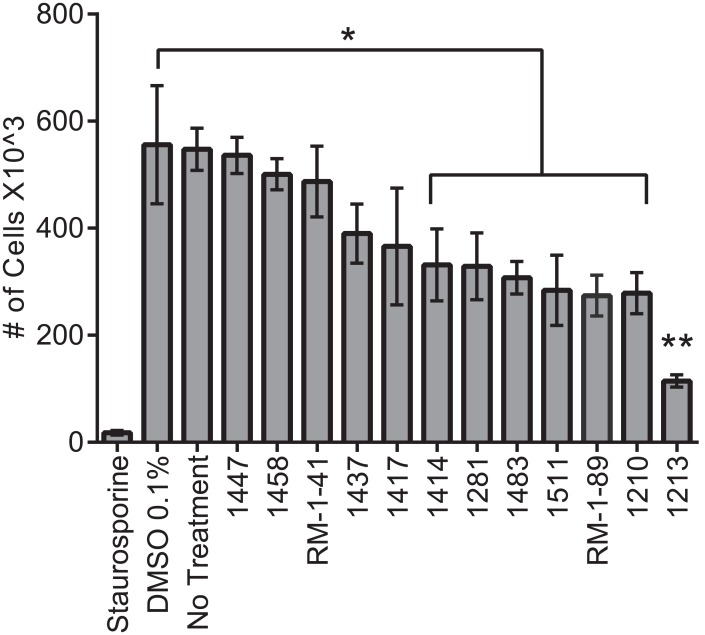
Bumped kinase inhibitors impact *Giardia* trophozoite growth. A subset ~10% of the existing BKI library was screened for impairment of *Giardia* growth at a concentration of 5 μM and results from selected compounds are shown. The general kinase inhibitor staurosporine reduced growth by 95%. Five bumped kinase inhibitors reduced growth by at least 50% (1483, 1511, Rm-1-89, 1210 and 1213.) The results are averaged from 3 biological replicates that were normalized to the control, DMSO 0.1%. Significance was evaluated by t-test, * = p<0.05, ** = p<0.01.

Our identification of genes encoding small gatekeeper kinases suggested the possibility that BKIs might affect *Giardia* trophozoites, the stage that colonizes the intestine. As a reference point, we assayed sensitivity of *Giardia* trophozoites to staurosporine, a broad spectrum kinase inhibitor that acts on small and large gatekeeper kinases. As shown in Fig 3, treatment with 5 μM staurosporine in 0.1% DMSO resulted in a 95% reduction in growth compared to vehicle alone or untreated cells. Next, we tested 36 compounds selected from a previously reported BKI library [26], for effects on growth at 5 μM concentration. Five of these compounds reduced growth by at least 50% compared to control cells treated with 0.1% DMSO. Compound 1213 reduced growth by 79% (Fig 3). Although, this BKI library was originally developed to explore selectivity for *Tg*CDPK1 and *Cp*CDPK1 over mammalian kinases [27], these results indicate that this class of compounds can effectively inhibit growth of *Giardia* trophozoites. The presence of multiple kinases with small gatekeeper residues and the ability of BKIs to inhibit *Giardia* growth suggest that BKIs could be developed as an alternative basis for treatment of giardiasis.

### Expression and subcellular localization of *Giardia* small gatekeeper kinases

**Fig 4:**
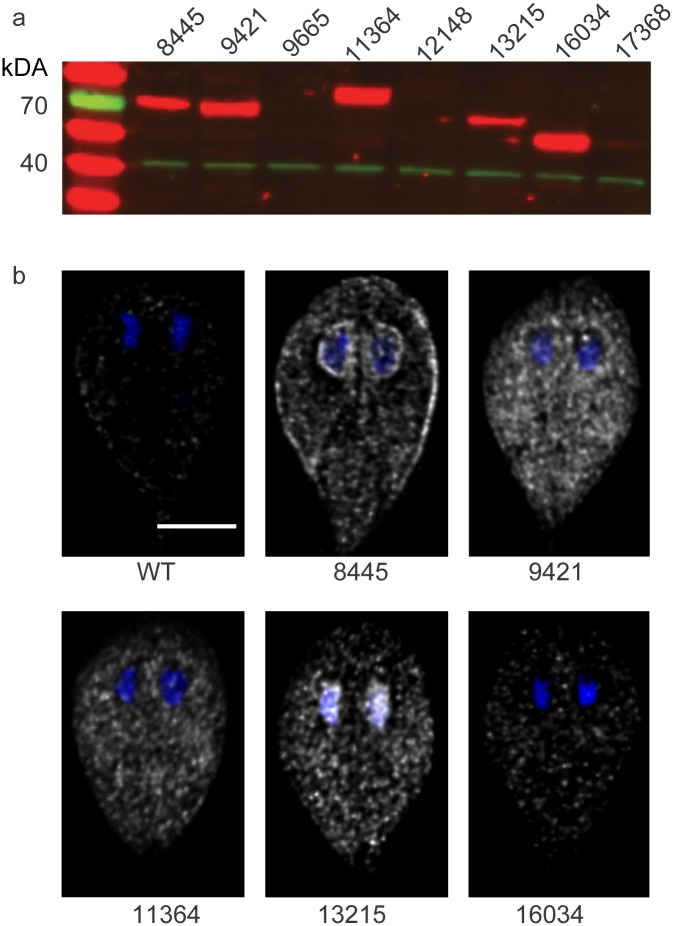
Integration of 3xHA tag allows visualization of protein expression and localization. **(a)** Western blot analysis showing kinases Gl50803_8445, Gl50803_9421, Gl50803_11364, Gl50803_13215 and Gl50803_16034 are expressed in trophozoites (red). Actin (green) was used as a loading control. **(b)** Immunofluorescence imaging of trophozoites expressing the designated protein: HA (grayscale) and DAPI (blue). Images were acquired and scaled identically. Representative images are shown from a minimum of 150 cells examined for each line. Scale bar = 5 μm.

Finding that BKIs can inhibit *Giardia* trophozoite growth established the priority to determine which kinases are critical for trophozoite proliferation and attachment. To accomplish this, we used an epitope tag to facilitate detection of each kinase [29]. We established seven cell lines, each with one specific kinase gene endogenously epitope-tagged with 3xHA [29]. Our integration constructs lack promoters and start codons; therefore, detection of protein products indicated successful integration into the genome with expression driven by the native promoter. Western blot analysis showed that five of the seven lines expressed detectable levels of proteins of the predicted molecular weight (Fig 4a).

Attempts to integrate two of the seven kinase genes (*Gl50803_9665* and *Gl50803_17368*,) in six separate trials, failed to yield lines that expressed detectable levels of the tagged protein as assayed by Western blotting. To assess further whether successful integration had actually occurred, we used PCR to assay the kinase locus for integration. As shown in S1 Fig, successful integration of constructs was achieved for both *Gl50803_9665* and *Gl50803_17368*. The absence of detectable protein expression may be due to low level of endogenous protein expression or more likely developmental regulation; therefore, these two were not carried forward for genetic analyses.

Using the same cell lines that were established for Western blotting, we analyzed cellular localization as a secondary method for detecting relative abundance. Using identical exposure and scaling conditions, the five 3xHA-tagged kinases: Gl50803_8445, Gl50803_9421, Gl50803_11364, Gl50803_13215 and Gl50803_16034 were localized by immunofluorescence microscopy. We observed strong signals for kinase Gl50803_8445 around nuclei and the cell perimeter and lower levels distributed throughout the cytosol. Strong signals for kinases Gl50803_9421 and Gl50803_11364 were detected throughout the cytosol. Cytosolic signals for kinase Gl50803_9421 were also observed but were relatively weak. Cytosolic and nuclear signals were enriched for kinase Gl50803_13215. Finally, punctate signals for kinase Gl50803_16034 were uniformly dispersed throughout the cytosol (Fig 4b). These data document differences in the overall distribution of small gatekeeper kinases, with three distinct patterns resolved by our microscopy thus far. The differences may indicate different cellular roles in the trophozoite stage.

### Functional importance of *Giardia* small gatekeeper kinases

**Fig 5:**
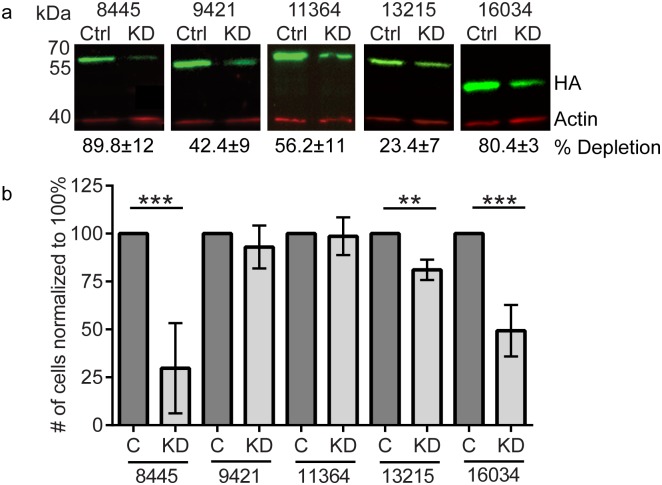
Kinase depletion interferes with growth. The five tagged-kinases shown to express in trophozoites were knocked down using anti-sense morpholinos. **(a)** Knockdown was measured and quantified by Western blot analysis, HA (green) and actin as loading control (red) **(b)** Cell growth was assessed after morpholino knockdown. Experiments were replicated a minimum of three times and significance was evaluated by t-test, * = p<0.05, ** = p<0.01, *** = p<0.001.

Following successful 3xHA integrated tagging of kinases we pursued a reverse genetics approach to test the relative importance of each kinase for trophozoite proliferation. Previous attempts to use RNAi for gene knockdown have been unsuccessful and gene knockouts in *Giardia* are yet to be accomplished due to the tetraploid nature of trophozoites [22]. Therefore, we chose to target the remaining kinases with anti-sense translation blocking morpholino oligomers. Treatment with 100 μM gene specific antisense-morpholino oligomers reduced protein levels by 89%±12 (Gl50803_8445), 42%±9 (Gl50803_9421), 56%±11 (Gl50803_11364), 23%±7 (Gl50803_13215), and 80%±3 (Gl50803_16034) compared to levels observed in cells with control morpholino oligomers (Fig 5a).

For kinases Gl50803_9421 and Gl50803_11364, we did not detect observable changes in growth despite appreciable depletion of protein levels, indicating that they likely may not be essential for trophozoite proliferation (Fig 5b and S2 Fig). Gl50803_13215 showed a significant, yet modest decrease in growth (Fig 5b). In contrast, depleting Gl50803_8445 and Gl50803_16034 resulted in 70% and 50% reduction in growth, respectively, compared to the control, 48 hours after morpholino treatment (Fig 5b).

### Thermal shift assays identify BKIs with high affinity to target kinases

**Fig 6:**
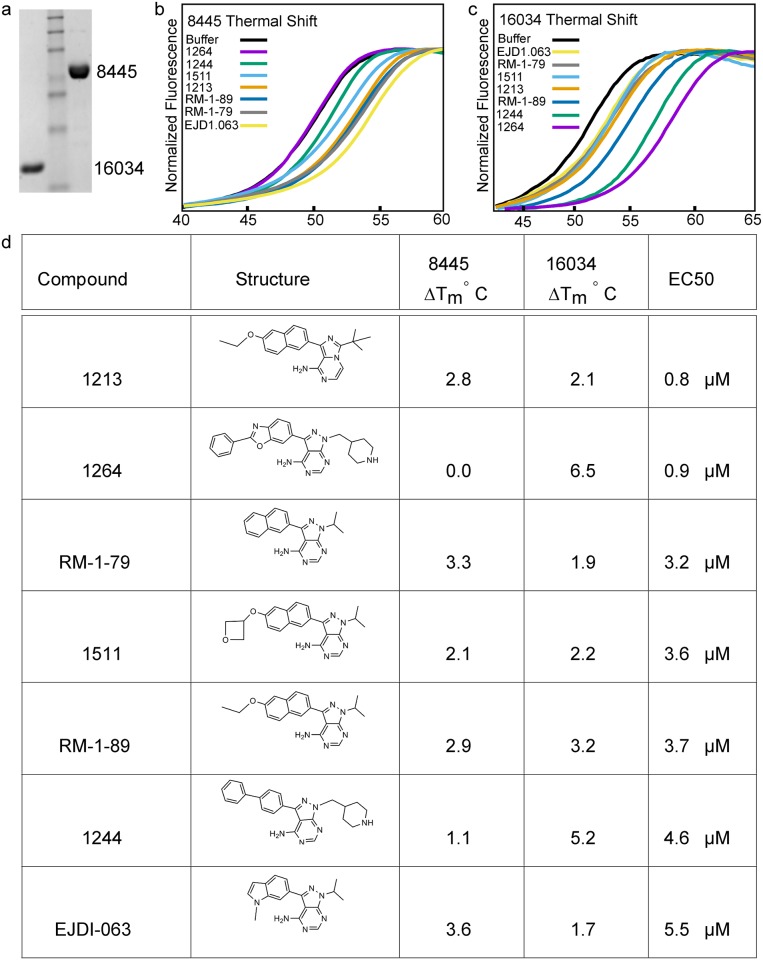
Thermal shift assays identify bumped kinase inhibitors that bind to purified proteins. Kinases Gl50803_8445 and Gl50803_16034 were purified from *E*. *coli*. **(a)** SDS-PAGE was used to estimate protein purity. Gl50803_8445 was 91% pure and Gl50803_16034 was 98% pure. Note that minor impurities are not expected to affect ΔTm determination. **(b, c)** Melting curves for the BKIS showing the largest ΔTm are shown for Gl50803_8445 (b) and Gl50803_16034 (c). **(d)** The name, chemical structure, ΔTm, and EC_50_ value obtained for 7 BKIs prioritized for phenotypic characterization of anti-growth activity (Also see S3 Fig).

Thermal shift assays (TSAs) quantify the change in the denaturing temperature of a protein due to the stabilizing presence of a bound small molecule, in this case bumped kinase inhibitors bound at the kinase active site. Denaturation is conveniently tracked by following increased fluorescence from a dye that associates with hydrophobic regions that are exposed as the protein denatures [40,41]. For a given protein target, the magnitude of the thermal shift induced by individual small molecules is roughly correlated with the binding affinity of that molecule [35]. This obviates the need to determine activation requirements and suitable substrates for in vitro kinase activity assays of individual target kinases. We used TSAs to rapidly screen the BKI library in order to prioritize a subset of them for individual characterization of anti-giardial activity. After cloning, expressing and purifying both Gl50803_8445 and Gl50803_16034 proteins, we were able to assess ~400 BKIs for interaction with each purified kinase (Fig 6). Two compounds (1264, 1244) induced large shifts (ΔTm > 5°) in the stability of target Gl50803_16034. No equivalently large shifts were observed for target Gl50803_8445, although several compounds gave ΔTm ≈ 3°. Notably the two compounds with the largest effect on Gl50803_16034 showed minimal effect on GL50803_8445, confirming the expectation that the BKI library compounds can exhibit specificity even among small-gatekeeper kinases. Library compound 1213, previously shown to be potent in suppressing trophozoite growth, induced a moderate ΔTm (2°–3°) in both targets. To see if the implied binding to an essential kinase would translate into *in vivo* activity, we selected 7 compounds with relatively large ΔTm for assessment of their phenotypic suppression of growth or attachment.

### Matching cell-active BKIs to their intracellular target kinase[s]

Any or all of the small-gatekeeper kinases we have identified may constitute a target for new drugs against giardiasis. All are likely to be susceptible to bumped kinase inhibitors, and indeed a single compound may act on more than one of these kinases. However, compounds are not expected to be uniformly potent against all of the kinases. While rigorous identification of the specific target kinases for all library compounds found to have anti-*Giardia* activity is beyond the scope of the current report, we performed an initial evaluation of the *in vivo* activity of seven BKIs for which a large thermal shift implied binding to either Gl50803_8445 or Gl50803_16034 (Figs 6d and S3). EC_50_ values for these compounds were determined from dose-response curves for trophozoite growth in culture, based on the total number of cells present 48 hours after introduction of inhibitor. Compound 1264 stands out as having both a sub-micromolar EC_50_ (0.9 μM) against *Giardia* trophozoites and a large induced thermal shift (6.5°) for kinase Gl50803_16034. We infer that GI50803_16034 is a primary target, although not necessarily the sole target, for 1264. The lower *in vivo* potency (EC_50_ 4.6 μM) for compound 1244, which also shows large ΔTm (5.2°) for this same target kinase, is unexpected but may be due to poor cellular absorption or metabolic degradation.

By contrast, compound 1213 shows somewhat greater *in vivo* activity (EC_50_ 0.80 μM) but shows only modest stabilization of either of the target kinases tested (ΔTm ≈ 3°). This suggests either that the primary target of 1213 is a different kinase or that its potency arises by modest inhibition of multiple targets.

Our initial search for small gatekeeper kinases was focused on identifying kinases most similar to TgCDPK1 due to an available library that could be used for this proof of concept study. Considering the variability of EC50 values relative to thermal shifts, we performed a more exhaustive search for small gatekeeper kinases, this time setting no threshold on similarity to TgCDPK1. Three additional NEK kinases were identified (GL50803_8152, GL50803_112518, GL50803_40904) and a putative threonine-gatekeeper CDC7 homolog (GL50803_112076). Although initial alignment of active site residues was less certain for sequences in this wider search, we confirmed the identity of the gatekeeper residue in GL50803_112076 by comparison with the known gatekeeper for CDC7 homologs with structures in the PDB. These four hits from the exhaustive search constitute possible additional targets for the activity of BKI compounds reported here.

**Fig 7:**
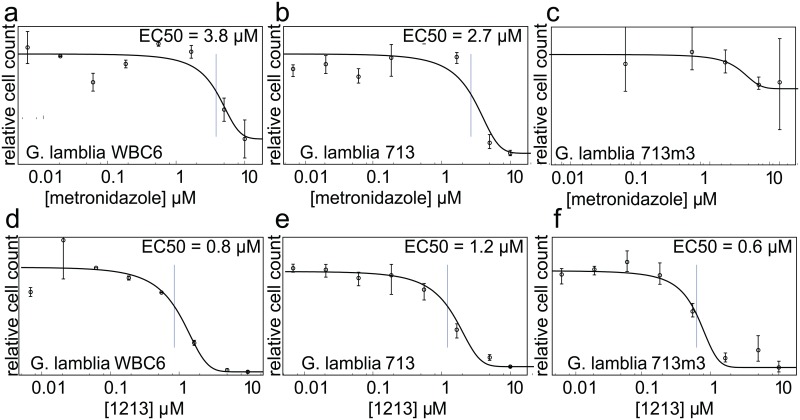
BKIs are effective against metronidazole-resistant *Giardia* cells. **(a)** Wild-type *G*. *lamblia* clone *WBC6* and **(b)** clone *713* are inhibited by metronidazole (EC_50_ of 3.8 μM and 2.7μM, respectively), but (**c)** metronidazole-resistant clone *713-M3* is not. Clones **(d)** *WBC6*, **(e)** *713* and **(f)** *713-M3* are each inhibited by BKI 1213 (EC_50_ of 0.8 μM, 1.2 μM, and 0.6 μM, respectively).

Note that whether the BKIs act through inhibition of a single target or through inhibition of multiple kinases, this mechanism is distinct from that of the existing anti-giardiasis drug metronidazole and chemically related alternatives. Therefore, cross-resistance is unlikely. This is confirmed by the observed equal potency of compound 1213 against both wild-type and metronidazole-resistant strains of *Giardia* (Fig 7).

### Kinase 8445 and 16034 are critical for cytokinesis and attachment

**Fig 8:**
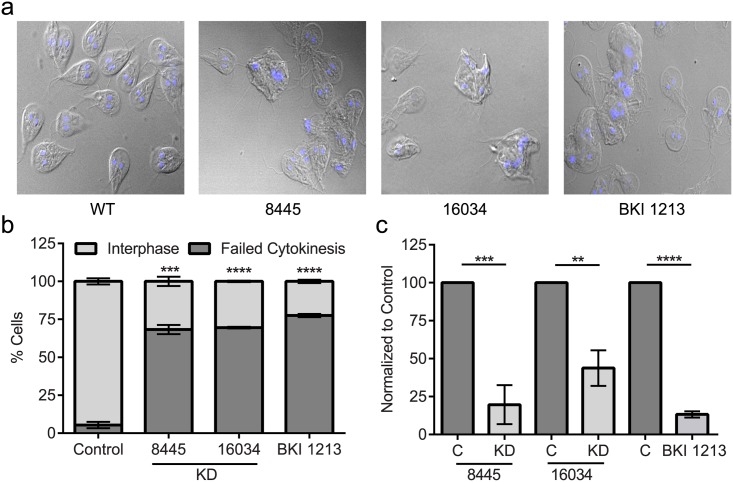
BKI 1213 mimics defects observed in knockdown of kinases Gl50803_ 8445 and Gl50803_16034. **a)** Fluorescent microscopy images of cells 48 hours after morpholino knockdown or treatment with BKI 1213 demonstrate a defect in cytokinesis compared to wild type control cells; cells complete nuclear division but not cytokinesis. **b)** Quantification of percent of cells that failed to complete cytokinesis. In each treatment, more than 67% of cells were stuck in cytokinesis; 68% of Gl50803_8445, 69.5% of Gl50803_16034 and 77.5% of cells treated with BKI 1213 were unable to complete cytokinesis. **c)** Attachment assays confirmed that when protein is depleted or cells are treated with BKI 1213, the ability for cells to attach is greatly reduced. Less than 20% of kinase 50803_8445 cells were able to maintain attachment, 43.8% of kinase 50803_16034 cells were able to maintain attachment and only 13% of cells treated with 5 μM BKI 1213 maintained attachment. Experiments were replicated a minimum of three times and significance was evaluated by t-test, ** = p<0.01, *** = p<0.001, **** = p<0.0001.

Given the critical role of kinase Gl50803_8445 and Gl50803_16034 in *Giardia* trophozoite growth, we analyzed the terminal phenotype of these kinases and found that depleting either kinase resulted in multinucleate cells indicating a block in cytokinesis (Fig 8). Quantification indicated 68% of l50803_8445 and 70% of Gl50803_16034 cells were blocked in cytokinesis. Compound 1213 induced a similar defective cytokinesis phenotype with slightly higher efficacy than knockdown of either kinase alone. This may reflect incomplete knockdown by morpholino treatment or an additive effect of chemical inhibition acting on multiple kinases.

Clinical giardiasis is caused by *Giardia* trophozoites attaching to the intestinal microvilli, colonizing the intestines and creating a barrier against nutrient absorption by the host. Additionally, histological tissue samples have indicated an increased production of mucus by host goblet cells and vacuolated epithelial cells in the mucosa [42,43], that likely reflect an effort to dislodge attached parasites. Therefore, kinases with a role in attachment, even indirectly, would be promising targets, as infection could be cleared without necessarily killing the parasites. An example of this pharmacological strategy is the demonstration that the anti-giardial activity of the isoflavone formononetin occurs by inducing rapid detachment of trophozoites from the intestine of infected mice [44]. In cell culture, *Giardia* attaches to the surface of the culture tube, with a small fraction of cells detached and freely swimming.

Therefore, we assessed the ability of trophozoites to remain attached 48 hours post-knockdown for the two kinases validated to be essential for growth. This was assayed by monitoring the ratio of cells that were free-swimming to those attached to the culture tube. We observed that the ability of cells to attach to the culture tube was substantially reduced following knockdown of Gl50803_8445 and Gl50803_16034 cells. We observed an 80% and 56% decrease in parasite attachment (Fig 8c) when we knocked down Gl50803_8445 and Gl50803_16034, respectively. In this assay, we simply poured off detached cells; maintaining attachment during intestinal peristalsis would likely present a greater challenge to the cells. These genetic experiments, performed at the population level with incomplete knockdown indicate that targeting kinases Gl50803_8445 and Gl50803_16304 with BKIs could be an effective strategy to clear a *Giardia* infection due to reduced attachment and proliferation. Indeed, treatment of trophozoites with compound 1213 led to an 87% decrease in parasite attachment.

## Discussion

### Previous phenotypic screening for anti-giardiasis activity

Several efforts to identify potential new anti-giardial therapeutics by phenotypic screening against large libraries of drugs or drug-like molecules have been reported previously. Tejman-Yarden et al [45] screened a library of 910 bioactive compounds including ~750 approved drugs. Of these, 56 compounds exhibited inhibition of *G*. *lamblia* growth and attachment at 10 μM, including 15 compounds with known anti-giardial activity and most notably the approved anti-arthritis drug auranofin.

Auranofin was reported to be active against *Giardia* trophozoites with an EC_50_ of 4–6 μM. Galkin, et al [46] found the most active compound in the LOPAC^1280^ library of pharmaceutically active compounds to be disulfiram (tetraethylthiuram disulfide), previously used in long-term treatment of alcoholism. They reported an EC_50_ of 0.9 μM against *Giardia* trophozoites. Disulfiram covalently attacks protein sulfhydryls in general, but the primary target in this case is a non-catalytic cysteine near the active site of *G*. *lamblia* carbamate kinase. Both of these potential leads, disulfiram and auranofin, overlap in part with the chemical action of existing nitroimidazole drugs including metronidazole in attacking protein sulfhydryl groups. Thus, they share a profile of side-effects and potential toxicity, although the specific sets of intracellular targets differ [47].

The use of phenotypic screening is generally viewed in contradistinction to molecular target-based approaches to drug discovery [48]. Identifying off-label activity of an approved drug through phenotypic screening has the obvious benefit of immediate clinical applicability if the compound has sufficient anti-parasite activity. On the other hand, it is not clear that either of the previously reported hits from anti-giardial screening offer much scope for follow-on chemical modification of the existing drug to improve selectivity or anti-giardial potency. This illustrates a common drawback of phenotypic screening, which is further exacerbated if the molecular target of a cell-active compound found by screening is not known, precluding structure-guided optimization of selectivity or potency.

### Success of combined structural genomics and targeted phenotypic screening

We have been able to combine the strengths of phenotypic screening and a structural approach to target-based lead discovery. Preliminary examination of the *G*. *lamblia* genome established that it contained eight coding sequences for kinase homologs with an atypically small (Gly/Ala/Ser/Thr) active site residue at the gatekeeper position. It is important to note that these sequences were not selected on the basis of sequence homology either to each other or to previously characterized drug targets. Instead they were selected because they are predicted to share a specific unusual structural feature at the putative active site. We then conducted an initial targeted phenotypic screen for anti-giardia activity in a representative subset of compounds drawn from a library designed to target exactly this shared structural feature, confirming that the library was rich in cell-active compounds.

Follow-up TSA screening of the entire library against purified kinases Gl50803_8445 and Gl50803_16034 highlighted specific library compounds with high implied affinity for those targets. Next, we showed that library compounds with high implied affinity for Gl50803_16034 exhibited EC_50_ ≤ 1 μM against *Giardia* trophozoites, confirming the success of this approach to lead discovery.

It is quite possible that the library compounds selected for high-implied affinity for Gl50803_16034 also bind others of the identified small-gatekeeper kinases not yet individually characterized. This does not diminish their potential as leads for anti-giardia drug design. It is formally possible that they also hit some unknown protein target, but we consider this unlikely based on prior characterization of compounds from this BKI library as being non-cytotoxic to mammalian cell lines and having low activity against a panel of larger gatekeeper kinases [26,31,44].

### Concluding remarks

Here we have shown that a set of kinases in *Giardia* possess an unusual structural feature, a small gatekeeper residue, which is a promising target for the development of highly selective inhibitors. Knockdown of target proteins identified two kinases with strong growth, attachment and cytokinesis defects; the cells appear to have completed nuclear division but never completely divided. As a result of the cytokinesis defect, growth is inhibited and a high percentage of cells are unable to maintain attachment presumably due to gross morphological defects impeding the function of the ventral adhesive disc [1,49]. Compounds from a BKI library originally designed to target *T*. *gondii* CDPK1 were able to phenocopy the results observed after kinase knockdown. In particular, exposure to BKI 1213 induced defects in cytokinesis similar to those produced by kinase knockdown as observed by fluorescence microscopy. Such a severe defect hinders the cells ability to attach and creates the potential to clear an infection from the intestines. Collectively, our results suggest that *in vivo* use of BKIs may provide an alternative treatment for giardiasis. Importantly, we have shown that BKIs can inhibit the growth of metronidazole-resistant *Giardia* due to their entirely different mechanism of action. Moreover, BKIs are not expected to have the problematic side effects associated with the free radical production by metronidazole and related drugs. Therefore, BKIs are an attractive starting point to find an alternative treatment for giardiasis.

## Supporting Information

S1 Table PCR primers, morpholino oligo sequences and PCR primers to verify integration of *Gl50803_9665* and *Gl50803_17368*. (EPS)

S1 Fig (a) Diagram of vector integration strategy (b) PCR assay to validate integration of 3xHA epitope tag at each kinase, *Gl50803_9665* and *Gl50803_17368*. (EPS)

S2 Fig Growth curves of kinases after knockdown vs morpholino standard control. (EPS)

S3 Fig EC_50_ of BKI inhibition of *Giardia* trophozoite growth. (EPS)
